# Electrocortical signatures of attentional bias toward subliminal and supraliminal socially negative words in social anxiety

**DOI:** 10.3389/fpsyt.2025.1506516

**Published:** 2025-02-13

**Authors:** Shuzhen Gan, Yanglong Cai, Weijun Li

**Affiliations:** ^1^ Shanghai Changning Mental Health Center, Affiliated Mental Health Center of East China Normal University, Shanghai, China; ^2^ Shanghai Mental Health Center, Shanghai, China; ^3^ The School of Psychology and Cognitive Science, East China Normal University, Shanghai, China; ^4^ Research Center of Brain and Cognitive Neuroscience, Liaoning Normal University, Dalian, Liaoning, China; ^5^ Key Laboratory of Brain and Cognitive Neuroscience, Dalian, Liaoning, China

**Keywords:** social anxiety, attentional bias, attentional orienting, attentional disengagement, N2pc, parietal P3, consciousness

## Abstract

**Background:**

Previous research has demonstrated that abnormal attentional bias toward social threats at different processing stages is pivotal for the development and maintenance of social anxiety. However, the temporal property and the neural indicators of this bias are still open to clarification. The present study employed event-related potential (ERP) methodology to investigate the attentional bias toward social threats at the early preconscious and later controlled processing stages, along with associated electrocortical indicators.

**Methods:**

Socially or non-socially negative words paired with neutral ones were presented subliminally and supraliminally in two dot-probe tasks, respectively. Twenty-six participants with high level of social anxiety (high SA) and twenty-four participants with low level of social anxiety (low SA) completed the tasks.

**Results:**

The results revealed that, compared to the low SA group, the high SA group specifically showed a significant N2pc in response to subliminal socially negative words, and the amplitude tended to correlate with anxious severity. Additionally, the high SA group exhibited greater amplitudes of parietal P3 in response to incongruent probes than congruent ones following both subliminal and supraliminal socially negative words.

**Conclusion:**

These results indicate that abnormal attentional bias of social anxiety includes both early preconscious attentional orienting to social threats and subsequent difficulty disengaging from conscious and unconscious social threats, as indexed by N2pc and parietal P3 components, respectively. Our study may hold clinical significance by providing electrophysiological markers for assessing the cognitive symptoms of social anxiety.

## 1 Introduction

Social anxiety is characterized by excessive fear and avoidance of social situations in which individuals worry about being scrutinized and receiving negative social feedback, such as negative evaluations, humiliation, and rejection due to their performance (1). The cognitive-behavioral model of social anxiety emphasizes the role of attentional bias toward external potential social threats and internal cues, including physiological responses and negative thoughts about oneself, in the development and maintenance of symptoms (2). It has been proposed and widely demonstrated that attentional bias in anxious disorders leads to hypersensitivity and elaborative processing to threats, which reinforces symptoms (3, 4). However, the mechanism and related neural biomarkers of the abnormal attentional bias of social anxiety are still open to clarification.

A commonly used paradigm to examine attentional bias is the dot-probe task (5, 6). In this task, a pair of stimuli usually consisting of a threatening stimulus and a neutral stimulus are displayed simultaneously in two different spatial locations on the screen as cues. Afterward, a probe appears in one of the two locations; the probes replace threatening stimuli are “congruent”, and those replace neutral stimuli are “incongruent”. Participants are required to respond to the probe as accurately and quickly as possible. Attentional bias is indexed by faster responses to congruent probes than incongruent ones. This is based on the assumption that the response would be facilitated and faster to the probe presented in the location that was attended to. The subcomponents of attentional bias, vigilance, avoidance, and disengagement difficulty, are further identified when a baseline condition, with stimuli pairs consisting of two neutral stimuli, is included (7). Compared to the baseline condition, faster responses to congruent probes appearing in the threat location indicate vigilance (i.e., fast attentional orienting), whereas slower responses indicate avoidance (i.e., slow attentional orienting); slower responses to incongruent probes appearing in the neutral location indicate disengagement difficulty.

Using the dot-probe paradigm, documented studies have revealed that attentional bias in anxiety disorders occurs at different stages of stimulus processing, with the exposure time of stimuli acting as a moderator (8). When using a short presentation duration (e.g., ≤100 ms), socially anxious individuals showed vigilance toward negative faces and words (9, 10). Additionally, when threatening stimuli are presented in a subliminal and mask condition, vigilance is consistently observed (11, 12). This is a preconscious processing bias which is stimulus-driven and does not depend on awareness; and this bias is considered as a crucial psychopathological factor for anxiety disorders (4). Studies have demonstrated the preconscious attentional bias in anxiety and social anxiety, and it robustly predicts severity of symptoms (11, 13). In addition, socially anxious individuals also exhibit difficulty in disengaging from threats even if they are task-irrelevant (14, 15), and it might due to the deficient ability of attentional control (16). Disengagement difficulty is commonly observed in supraliminally presented stimuli (17) from brief to long exposures (e.g., from 100 ms to 500 ms) in anxiety disorders (18–20). Recently, a neuroimaging study found that when angry faces were presented subliminally (e.g., 17 ms), behavioral inhibition (BI) children showed greater activation in the cerebellum in incongruent trials than congruent ones, which may suggest disengagement difficulty (21). It remains unclear whether the disengagement difficulty in social anxiety depends on the conscious perception of threats. Using supraliminal and long-time presentations (e.g., ≥500 ms), the strategic avoidance is usually found which is used to prevent processing threats to reduce fear or anxious feelings. This strategy has been found in both healthy controls (22) and patients (23–25) and might not be disorder-specific.

Although many studies have demonstrated multiple attentional biases in anxiety disorders and tried to reveal their temporal properties, inconsistent findings have been found. For instance, with a long exposure, sustained attention engagement (26, 27), absence of avoidance (28) and disengagement difficulty (29, 30), or none of any attentional bias were reported (9, 31, 32). This discrepancy might be related to various factors, including the reference stimulus paired with the threatening stimulus (i.e., angry faces paired with neutral faces or objects), whether situational anxiety was induced (6), etc. An important factor might be that attentional bias indexed by the reaction time to probes is not a suitable measure when stimuli are presented with a long duration. The RT to probes is an outcome of the mixture of multiple processes from the cues onset to probe offset, which might include early stimulus-driven automatic processes and later strategic controlled processes. Thus, the transient processes and the change of attentional patterns at different stages, which might be abnormal in anxiety, were not effectively measured. This confounding can be circumvented by employing the method of event-related potentials (ERPs), which has a high temporal resolution and provides a continuous measure to describe the attentional patterns that unfold over time. Previous studies have demonstrated that compared to RT indicators, ERP components more reliably indicate attentional biases of social anxiety with high internal consistency (33).

In the dot-probe task, ERPs time-locked to the cues and probes are analyzed to indicate attentional bias at early and later stages of stimulus processing, respectively. A commonly used ERP component time-locked to the cues is N2pc, which reflects selective spatial attention and engagement to salient stimuli and task-relevant stimuli (34, 35). It is a difference wave that shows more negative reflection in the contralateral electrodes than in the ipsilateral electrodes relative to the target location. Its latency is approximately 180-300 milliseconds after the onset of the cues, with the maximum amplitude appearing in the parieto-occipital electrodes (e.g., PO7/PO8) (34, 36). Research has shown that N2pc can indicate abnormal attentional bias in social anxiety. For example, individuals with high social anxiety exhibit enhanced N2pc amplitude to emotional faces (37), and the amplitude of N2pc reliably predicts the severity of symptoms (33). However, these studies all employed supraliminal threats, so it is still unclear whether N2pc can indicate attentional bias to unconscious threats in social anxiety. Moreover, the role of awareness in N2pc generation is still debated. Some studies have found that N2pc can indicate attentional capture by unconscious and masked self-faces (38), while other studies suggest that N2pc relies on awareness and is not elicited by subliminal stimuli (39). This discrepancy needs further investigation.

For the ERP components time-locked to the probes, the centro-parietal P3 (also termed as P3b) in 300-500 milliseconds after target onset is usually used to index controlled processes such as attention shift at a later stage (40–42). It can indicate top-down modulation of attention and resource allocation according to the task goal, and its amplitude varies with the effort devoted (41). In clinical samples, studies have demonstrated abnormal P3 effect elicited by probes. For instance, compared to healthy controls, depressive patients showed higher P3 amplitude to congruent probes than incongruent probes to negative faces (43), spider phobia patients exhibited larger P3 amplitude to congruent probes than incongruent ones specifically to spider images (44). These P3 effects elicited by probes is considered to reflect sustained attention to and disengagement difficulty from threats. In anxiety disorders, one study examined the attentional disengagement from test-related words in test-anxious individuals, and no effect was found on probe-elicited P3 (45). Whether centro-parietal P3 for probes can indicate abnormal attention modulation and disengagement difficulty of social anxiety is still unclear, whereas, this will provide evidence of the attentional bias at the later stage of threat processing in social anxiety.

In this study, socially negative words and non-socially negative words paired with neutral ones were presented subliminally (20 ms) (46) and supraliminally (500 ms) separately through two dot-probe tasks. Participants with high and low levels of social anxiety completed these two tasks, and their EEG data were collected. We analyzed the N2pc to word pairs and the centro-parietal P3 to probes to indicate attentional biases in the early and later stages of threat processing. Our research aims are (1): To reveal the abnormal early attentional orienting and later attentional disengagement in social anxiety through N2pc and centro-parietal P3, and explore their associations with symptom severity; (2) To determine whether ERP indicators can sensitively reflect attentional bias toward consciously and unconsciously perceived threats in the early and later stages of processing. We believe that investigating these issues will help depict the attentional patterns unfolding over time and provide valid electrophysiological markers for the cognitive symptoms of social anxiety.

## 2 Materials and methods

### 2.1 Participants

College students were recruited from Liaoning Normal University, and all participants completed the Liebowitz Social Anxiety Scale (LSAS). Students with scores lower than 30 were selected into the low social anxiety (SA) group, and those with scores higher than 60 were selected into the high SA group (47, 48). Ultimately, 50 students agreed to participate in the EEG experiment, with 26 in the high SA group and 24 in the low SA group. The sex and age were balanced between the two groups (sex: $\chi^{2}$ = 1.98, *p* > 0.05; age: t (48) = 0.84, *p* > 0.05). The demographic data of the high and low SA groups are displayed in **Table 1**.

**Table 1:** Mean and SDs (in parentheses) for the demographics and self-reported scores of questionnaires in high and low SA participants, and the statistic results of the group differences.

	High SA (n=26)	Low SA (n=24)	t
Male: Female	6:20	10:14	
Age	21.77 (2.05)	22.38 (3.09)	0.84
LSAS	80.58 (11.82)	19.42 (5.98)	22.79***
SADS	18.12 (4.48)	5.38 (6.09)	8.47***
State-STAI	49.19 (10.65)	34.88 (11.05)	4.66***
Trait-STAI	52.04 (8.92)	35.92 (9.07)	6.33***

High SA, the group with high level of social anxiety; Low SA, the group with low level of social anxiety; LSAS, Liebowitz Social Anxiety Scale; SADS, Social Avoidance and Distress Scale; State-STAI, State Anxiety subscale of State-Trait Anxiety Inventory; Trait-STAI, Trait Anxiety subscale of State-Trait Anxiety Inventory; ***p < 0.001.

All participants were right-handed, had normal or corrected-to-normal vision, and no history of substance addiction such as drug and alcohol. They were also required to have no current severe physical diseases and neurological disorders, and no medication use within the month before experiment. Participants were paid 10 RMB for completing the questionnaires and an additional 80 RMB for completing the EEG experiment. The Ethics Committee of Liaoning Normal University approved this study (protocol code LL2024137). Before the experiment, all participants were provided with and signed an informed consent in accordance with the Declaration of Helsinki.

### 2.2 Questionnaires

Before the experiment, all participants completed the Liebowitz Social Anxiety Scale (LSAS), Social Avoidance and Distress Scale (SADS), and State-Trait Anxiety Inventory (STAI). The average scores are shown in **Table 1**.

The LSAS was used to measure the severity of social anxiety (49). This scale contains 24 statements describing different social situations, and participants rated their anxious feelings and their avoidance behaviors separately for each item. The total score of the LSAS ranges from 0 to 144, with higher scores indicating greater severity of social anxiety.

The SADS assesses two aspects of social anxiety: the tendency to avoid social situations and the experience of negative feelings such as anxiety or discomfort when interacting with others (50). It consists of 28 true-false items with a total score range of 0-28. Higher scores indicate greater avoidance and distress in social situations.

Finally, the STAI was used to separately measure state anxiety and trait anxiety with two subscales (51). The former evaluates a temporary and current anxious state that might be elicited by the present situation, while the latter evaluates a stable anxious propensity that people generally feel. Each subscale consists of 20 items with a score range of 20-80, with higher scores indicating greater symptoms of state or trait anxiety.

### 2.3 Stimuli

Three types of Chinse two-character words—non-socially negative, socially negative, and neutral words—frequently used in daily life were selected. The socially negative words include words describing the hostile attitudes or behaviors toward others, as well as words reflecting a poor performance, appearance and feelings about oneself in social interactions. The non-socially negative words and neutral words have nothing to do with social interactions or situations. Thirty college students were recruited to rate the words’ emotional valence and arousal on a 9-point Likert scale, ranging from completely unpleasant (1) to completely pleasant (9) for valence, and from completely calm (1) to completely excited (9) for arousal. Negative words with valence higher than 4 and neutral words with valence outside the 4-6 range were deleted. Finally, 40 non-socially negative words, 40 socially negative words, and 120 neutral words were used. Each negative word was then paired with a neutral word, yielding 40 non-socially negative-neutral (NN) word pairs and 40 socially negative-neutral (SN) word pairs. The remaining neutral words were paired with each other, resulting in 20 neutral-neutral (N) word pairs. According to the rating results, compared to the matching neutral words, both non-socially negative and socially negative words were rated as more unpleasant [non-socially negative vs. neutral: t (78) = 18.20, *p* < 0.001; socially negative vs. neutral: t (78) = 32.08, *p* < 0.001] and more arousing [non-socially negative vs. neutral: t (78) = 13.62, *p* < 0.001; socially negative vs. neutral: t (78) =20.08, *p* < 0.001]. The socially negative words were more unpleasant [t (78) = 2.93, *p* = 0.01], and were also more arousal than non-socially negative words (t (78) = 2.85, *p* = 0.01)]. The mean valences and arousals of socially and non-socially negative words, their paired neutral words, and the neutral words in N word pairs were shown in **Table 2**. In addition to the emotional dimension, we further examined whether word frequencies matched across different types of words. We gained the word frequencies from the SUBTLEX-CH corpus (52), and the frequencies of the socially and non-socially negative words were matched with neutral words [socially negative vs. neutral: t (78) = 1.40, *p* > 0.05; non-socially negative vs. neutral: t (78) = 1.83, *p* > 0.05]. The socially and non-socially negative words were also matched on frequency [t (78) = 1.79, *p* > 0.05]. All the words used in the experiment are shown in **Supplementary Table S1**.

**Table 2:** Mean valences and arousals and SDs (in parentheses) for each category of words.

	SN		NN		N
	Negative	Matched neutral	Negative	Matched neutral	
Valence	2.47 (0.37)	5.04 (0.35)	2.82 (0.67)	5.07 (0.40)	5.10 (0.36)
Arousal	6.26 (0.58)	2.96 (0.85)	5.71 (1.06)	2.87 (0.78)	2.97 (0.78)

SN, socially-negative word pairs; NN, non-socially negative word pairs; N, neutral-neutral word pairs; Negative, the negative word in the pair; Matched neutral, the neutral word in the pair.

### 2.4 Task design and procedure

A mixed design of group (high SA, low SA) × word (NN, SN) × probe (congruent, incongruent) was employed, with word and probe as within-subject factors and group as the between-subject factor.

**Figure 1:**
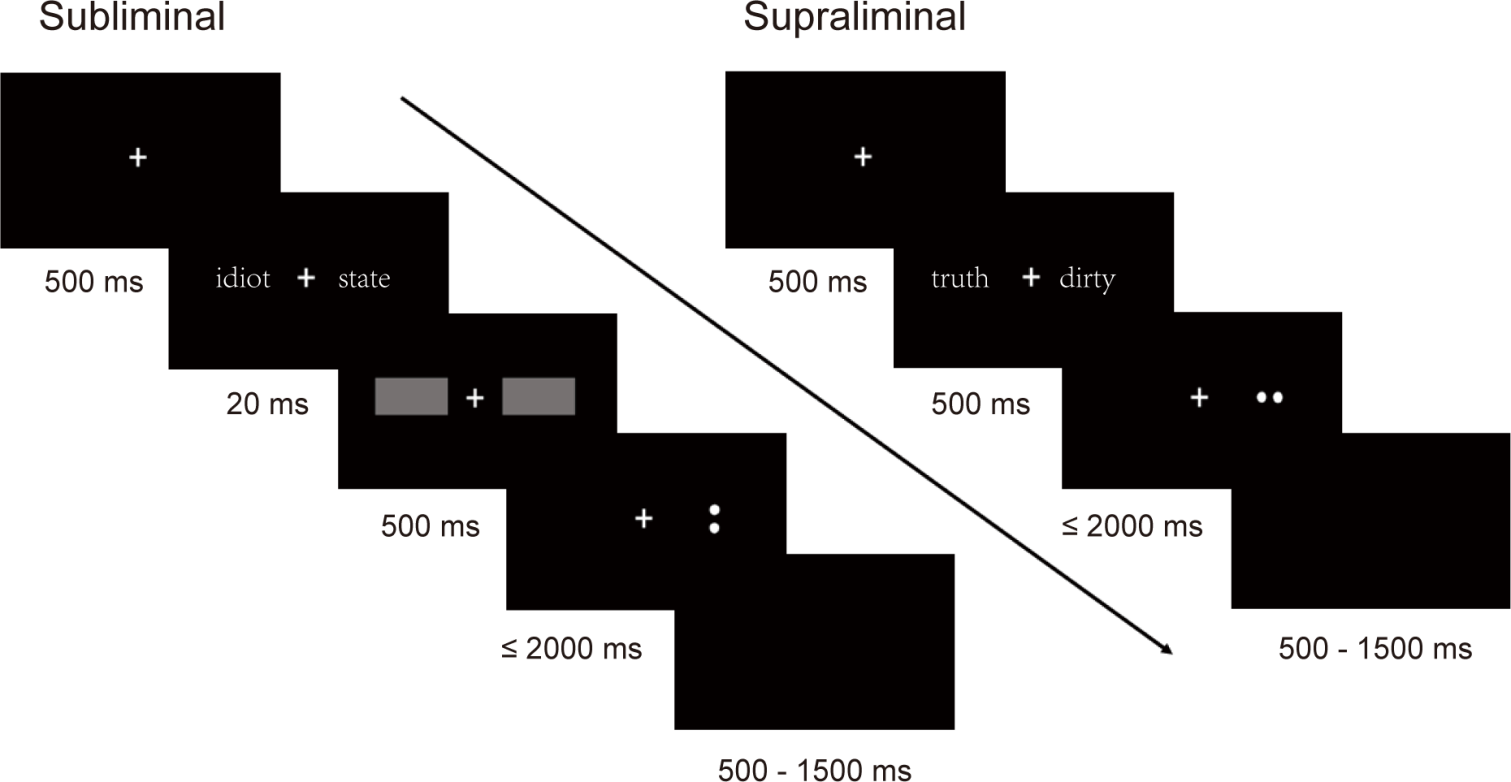
The presentation order and duration of an example trial in subliminal and supraliminal tasks.

Two modified dot probe tasks were used to assess attentional bias toward subliminally and supraliminally presented word pairs, respectively. In a typical trial (**Figure 1**), a “+” was first presented on the central screen for 500 ms, followed by a word pair with each word appearing on the left or right side of the “+”. In the supraliminal task, the display duration of the word pair was 500 ms. In the subliminal task, the word pair was displayed for 20 ms and then quickly replaced by a mask consisting of two gray rectangles for 500 ms. After that, the probe, either an upright colon “:” or a rotated horizontal colon “.”, appeared in one of the locations where the negative or neutral word had been. Participants were required to determine whether colon’s direction was upright or horizontal by pressing the *n* or *j* key on the keyboard using their index finger or middle finger, respectively. For half of the participants, the *n* key indicated “upright”, the *j* key indicated “horizontal”. While for the other half of participants, the keys assignments were reversed. Participants were asked to respond accurately and quickly within 2000 ms. The average jitter inter-trial interval (ITI) was 1000 ms (range: 500-1500 ms).

The locations of the negative words in the NN and SN word pairs were randomized, with half on the left and half on the right side of the “+”. The probe appeared randomly and equally in either of the two locations. There were five probe conditions: congruent probe following NN word pair (CON-NN), congruent probe following SN word pair (CON-SN), incongruent probe following NN word pair (INCON-NN), incongruent probe following SN word pair (INCON-SN), and baseline condition, probe in the random left or right location following N word pair (N). A total of 200 trials were included, with 40 trials in each condition, and each word pair was presented twice in the task. The order of these trials was pseudorandomized and kept consistent across all participants.

Participants were led into a dimly-lit and sound-proof room after informed consent and completed the subliminal task first, followed by the supraliminal task. They were seated in front of a computer screen at a distance of 70-80 cm. All stimuli were presented on a 23-inch computer screen with a refresh rate of 60 Hz. The experimental procedure was programmed and presented using E-prime 3.0 (Psychology Software Tools, Inc.). Before the experiment, participants practiced for 20 trials to familiarize themselves with the two tasks. The experiment was divided into 8 sessions, with 4 sessions for each task, and participants could rest between sessions. The entire experiment took 30 minutes to complete. The percentage of correct responses (accuracy) and reaction times (RTs) were recorded.

### 2.5 Awareness check task

We checked the manipulation of subliminal and supraliminal presentations by examining participants’ awareness in the two tasks. All word pairs presented in the experiment (40 SN, 40 NN and 20 N) and additional 60 new neutral-neutral (N) word pairs were used and constituted 80 negative-neutral and 80 neutral-neutral word pairs. Half were presented for 500 ms, while the other half were presented for 20 ms, followed by a pair of gray rectangles for 500 ms as a mask.

We recruited 22 college students to complete the awareness check task. Participants were required to determine within 2000 ms whether a word pair contained a negative word by key pressing. For half of the participants, pressing the *n* key indicated “yes”, and pressing *j* key indicated “no”. For the other half, the keys indicated opposite meanings. Then, participants rated their confidence level regarding their judgment on a 5-point Likert scale ranging from “not confident at all” (1) to “very confident” (5). The experiment consisted of 160 trials, with the order of subliminal and supraliminal trials randomized.

One-sample t-tests revealed that the accuracy of judgments for subliminally presented word pairs (M = 0.52, SD = 0.11) was not different from the random chance [t (21) = 0.98, *p* = 0.34], whereas the accuracy for supraliminally presented word pairs (M = 0.88, SD = 0.08) was significantly higher than the random chance [t (21) = 22.15 *p* < 0.001]. The RTs for subliminal word pairs (M = 916.04, SD =191.35) were significantly longer than that for supraliminal ones (M = 625.53, SD = 125.96) [t (21) = 9.39, *p* < 0.001]. Additionally, confidence ratings for judgments of subliminal presentations (M = 2.39, SD = 0.58) were significantly lower than that for supraliminal ones (M = 4.63, SD = 0.31) [t (21) = 16.23, *p* < 0.001]. We divided the confidence ratings for subliminal word pairs into two groups based on judgment accuracy: one for correct judgments and the other for incorrect judgments. Results showed no difference in confidence ratings between the correct responses (M = 2.40, SD = 0.46) and incorrection responses (M = 2.23, SD = 0.56) [t (21) = 1.91, *p* = 0.07].

In summary, these results demonstrated the effectiveness of our manipulation of subliminal and supraliminal presentations. Compared to the supraliminally presented word pairs, participants judged the emotionality of subliminally presented word pairs at a random level, required longer response times, and had lower confidences in their decisions. Their equal confidence levels for correct and incorrect responses to subliminal word pairs further supported the notion that their responses were at the unconscious level.

### 2.6 Electroencephalography data recording and processing

Scalp electrical activity was recorded using an elastic scalp cap with 62 Ag/AgCl electrodes placed according to the 10-20 system (ANT Neuro EEGO Inc., Germany). Additionally, data from two electrodes placed on the left and right mastoids were collected. EEG data were referenced online to an electrode placed between Cz and Pz, amplified at a sampling rate of 500 Hz, and low-pass filtered at 100 Hz. The impedances of all electrodes were kept below 5 kΩ.

Offline analysis used EEGLAB (53), Fieldtrip (54), and in-house code implemented in the MATLAB environment (The MathWorks Inc., Natick, MA, USA). To analyze word pair-elicited N2pc, we re-labelled the conditions of trials in the continuous EEG dataset according to the location of negative word in the pair. The negative words appeared in the left and right visual fields were separately labelled as ‘SN-left’ and ‘SN-right’ for SN word pairs, and ‘NN-left’ and ‘NN-right’ for NN word pairs. Then, we performed preprocessing. EEG data from scalp electrodes were re-referenced to the average of the two mastoids, band-pass filtered at 0.1-30 Hz. Bad blocks were rejected through visual inspection, and independent component analysis (ICA) was then conducted to remove ocular artifacts (eyeblinks and saccades) (53). Word pair-locked epochs were then extracted from 200 ms before to 500 ms after word pair onset, using the averaged data from the pre-stimulus 200 ms as a baseline. Epochs with excessive physiological artifacts exceeding ±100 μV were excluded. Afterward, data from the two posterior lateral electrodes, PO7 and PO8 were selected and analyzed (34, 36). The contralateral condition data were obtained from the two electrodes when SN and NN negative words appeared in their contralateral visual field, i.e., PO7 for ‘SN-right’ and ‘NN-right’ trials, and PO8 for ‘SN-left’ and ‘NN-left’ trials. The ipsilateral condition data were obtained from the two electrodes when negative words appeared in their ipsilateral visual field, i.e., PO7 for ‘SN-left’ and ‘NN-left’ trials, and PO8 for ‘SN-right’ and ‘NN-right’ trials. Trials within contralateral and ipsilateral conditions were averaged separately to obtain ERPs for SN and NN word type for each participant. The numbers of trials within contralateral and ipsilateral conditions for averaging were 80. After artifact rejection, the mean numbers of epochs remaining for ERP averaging for each type of word pairs, task and group ranged from 78 to 80, as shown in **Table 3**.

**Table 3:** Mean numbers of trials and SDs (in parentheses) remaining for ERP averaging for word pair-elicited N2pc and probe-elicited P3 under each condition.

	High SA group		Low SA group	
	Subliminal task	Supraliminal task	Subliminal task	Supraliminal task
N2pc (Contralateral/Ipsilateral)				
SN	78.77 (1.75)	79.00 (1.36)	78.38 (3.05)	79.13 (2.21)
NN	79.08 (1.29)	79.08 (1.44)	78.71 (3.24)	79.38 (2.26)
P3				
CON-SN	36.65 (4.56)	37.54 (3.62)	37.04 (2.49)	38.04 (2.33)
INCON-SN	38.23 (3.33)	37.73 (4.17)	37.21 (3.24)	38.96 (1.71)
CON-NN	37.42 (4.01)	37.85 (3.75)	37.46 (3.35)	38.58 (2.06)
INCON-NN	37.58 (4.02)	38.54 (3.19)	37.88 (2.49)	38.17 (3.02)

SN, socially negative word pair; NN, non-socially negative word pair; CON-SN, congruent probe after socially negative word pair; INCON-SN, incongruent probe after socially negative word pair; CON-NN, congruent probe after non-socially negative word pair; INCON-NN, incongruent probe after non-socially negative word pair.

In addition to N2pc, we further analyzed sustained posterior contralateral negativity (SPCN) time-locked to word pairs to investigate the sustained attentional patterns toward socially and non-socially negative words. SPCN is an extension ERP of N2pc, occurring in the 350-650 ms time window or longer. Its amplitude is larger at electrodes contralateral to the target than at ipsilateral electrodes. SPCN is considered to reflect prolonged processing and maintenance in working memory to stimulus at the attended spatial location (55, 56). The conditions and electrodes for SPCN analysis were same as those for N2pc, so no additional processing was conducted specific for SPCN.

For the analysis of probe-elicited P3, original condition labels were used, including CON-SN, CON-NN, INCON-SN, INCON-NN. The preprocessing was identical to that for N2pc, except for the epoch segmentations. The epoch time windows ranged from 200 ms prior to word pair onset (i.e., 700 ms and 720 ms before the probe for supraliminal and subliminal trials, respectively) to 500 ms after the probe onset, with the averaged data from 200 ms before word pair serving as the baseline (57, 58). Waveforms from Cz and Pz were analyzed, because the centro-parietal P3 showed maximum amplitude at the midline electrodes (43, 59). Trials from these two electrodes were averaged separately within each condition. The mean numbers of epochs remaining for ERP averaging for each condition ranged from 36 to 39, as displayed in **Table 3**.

Early components (e.g., P100) time-locked to the probe were not analyzed because the probe was immediately after the word pair, making the early ERPs of probe were probably distorted by the ERP response elicited by the word pair.

### 2.7 Statistical analyses

#### 2.7.1 Behavioral data

To investigate attentional bias, both traditional and response-based computations were applied to analyze the behavioral data.

##### 2.7.1.1 Traditional computation

For the response accuracies to the probes, a group (high SA, low SA) × word (NN, SN) × probe (CON, INCON) Repeated-Measures (RM) ANOVA was performed separately for the subliminal and supraliminal tasks.

For the RTs to the probes, false responses were first removed, then correct responses with RTs exceeding three SD from the mean were also excluded. The remaining data were analyzed with group (high SA, low SA) × probe (CON-NN, CON-SN, N) and group (high SA, low SA) × probe (INCON-NN, INCON-ST, N) RM ANOVAs to investigate the attentional orienting and disengagement, respectively. Shorter RTs to CON compared to N indicate vigilance, longer RTs to CON compared to N indicate avoidance, longer RTs to INCON compared to N indicate difficulty disengaging from negative words (30). Additionally, planned t-tests examining orienting (CON vs. N) and disengagement (INCON vs. N) were performed separately for SN and NN words in each group. To reduce Type I errors in multiple comparisons, all p-values were Bonferroni-corrected. When the effects of orienting and disengagement were significant in both groups, the intensity of orienting calculated by N-CON and disengagement difficulty calculated by INCON-N were further submitted to group × word RM ANOVAs to examine group differences.

##### 2.7.1.2 Response-based computation

In the traditional computation of attentional bias, the RTs of congruent and incongruent trials are averaged and compared with that of baseline trials, which reveals a single attentional pattern for each participant. However, it has been proposed that participants often use different attentional patterns for consecutively presented stimuli, such as showing vigilance to one negative stimulus and avoidance to the next, possibly due to limited attentional resources and economizing processing (60, 61). To distinguish different attentional patterns that vary intra-individually, the response-based measurement was developed (60). For the attentional orienting, the individual RT from each congruent trial was subtracted from a reference, the mean RT of N trials, i.e., RT_N_ - RT_CON [Trial1, Trial 2, ……Trial n]_. Positive difference scores indicate fast orienting and negative scores indicate slow orienting; the averaged difference scores of fast and slow orienting trials indicate the absolute magnitude of vigilance and avoidance to negative words, respectively. Similarly, for the disengagement, the mean RT of N trials was subtracted from the individual RT from each incongruent trial, i.e., RT_INCON [Trial1, Trial 2, ……Trial n]_ - RT_N_. Positive difference scores indicate slow disengagement, and negative difference scores indicate fast disengagement; the average scores indicate the absolute magnitude of disengagement difficulty and faster disengagement from negative words, respectively. These intra-individual attentional patterns have been demonstrated to be sensitive to social anxiety (62).

The statistical strategy for the absolute magnitudes of these attentional patterns was similar to that for RTs in traditional attentional bias. Group (high SA, low SA) × orienting direction (fast orienting, slow orienting) × word (NN, SN) and group (high SA, low SA) × disengagement direction (slow disengagement, fast disengagement) × word (NN, SN) RM ANOVAs were conducted to examine the relative magnitude of different attentional patterns in orienting and disengagement, respectively (62). Planned t-tests were also performed to examine the relative magnitudes for each word type in each group. Group differences were further examined when the effects of orienting and disengagement direction were significant in both groups.

In addition to the absolute magnitudes calculated based on RTs, the frequency of each attentional pattern was also obtained to examine whether the frequency differed between fast and slow orienting, and between slow and fast disengagement, respectively. The trial numbers for fast/slow orienting and disengagement were counted and submitted to group × orienting × word and group × disengagement × word RM ANOVAs.

#### 2.7.2 ERP data

Based on visual inspection, the time windows for N2pc varied within 180-300 ms across different word-pair types and groups. To determine the specific time clusters, a data-driven analysis, Monte-Carlo permutation test (54) was employed. In this procedure, paired t-tests comparing the amplitudes of contralateral and ipsilateral conditions were conducted at each time point between 180 ms and 300 ms. Adjacent significant samples (p < 0.05) were clustered. The cluster-level statistic was then calculated by summing the t-values from each time point within a cluster. To control Type I errors, the permutation procedure was conducted. Data from the contralateral and ipsilateral conditions were merged and randomly assigned to two pseudo-conditions. Similar paired t-tests between these two pseudo-conditions were performed at each point, and cluster-level statistics were calculated. By permuting the data for 2000 times, we obtained a null distribution of cluster-level statistics. The values of cluster-level statistic obtained from the real conditions were then compared against the null distribution. When it was greater than the 97.5th percentile or less than the 2.5th percentile of the null distribution, the difference between contralateral and ipsilateral conditions was considered significant. When the N2pc effect was significant in both groups, further examination of group differences in N2pc amplitudes, calculated by subtracting the amplitudes of the ipsilateral condition from those of the contralateral condition, was performed using a similar permutation test.

To examine the SPCN, similar permutation tests as those used in N2pc were performed at each time point between 350-500 ms to identify the temporal clusters, where contralateral condition had larger amplitudes than ipsilateral condition.

Temporal clusters of probe-related P3 effect were also obtained via permutation tests comparing congruent and incongruent condition within 300-500 ms at Cz and Pz electrodes (43). When the probe effect was significant in both groups, further examination of the group difference in the intensity of the probe effect, indicated by the difference in the amplitude between INCON and CON conditions, was conducted.

Finally, we examined how the N2pc, SPCN amplitudes and the intensity of P3 effect related to psychometric measures and behavioral attentional patterns using Pearson correlation analyses. Since we concerned about the processing bias toward social threats, the N2pc, SPCN and P3 magnitudes used in the correlation analysis were calculated from significant temporal clusters for SN word pairs.

## 3 Results

### 3.1 Behavioral results

#### 3.1.1 Traditional computation

For probe accuracy, no main effects or interactions were observed in the subliminal task (*ps* > 0.05). In the supraliminal task, participants responded more accurately to congruent probes than to incongruent ones [F (1,48) = 5.23, *p* = 0.03, $\eta_{p}^{2}$ = 0.10]. Additionally, the effect of word type was marginally significant [F (1,48) = 3.87, *p* = 0.06, $\eta_{p}^{2}$ = 0.07], with participants tending to respond more accurately to SN word pairs than to NN word pairs. No other effects or interactions were found (*ps* > 0.05).

**Figure 2:**
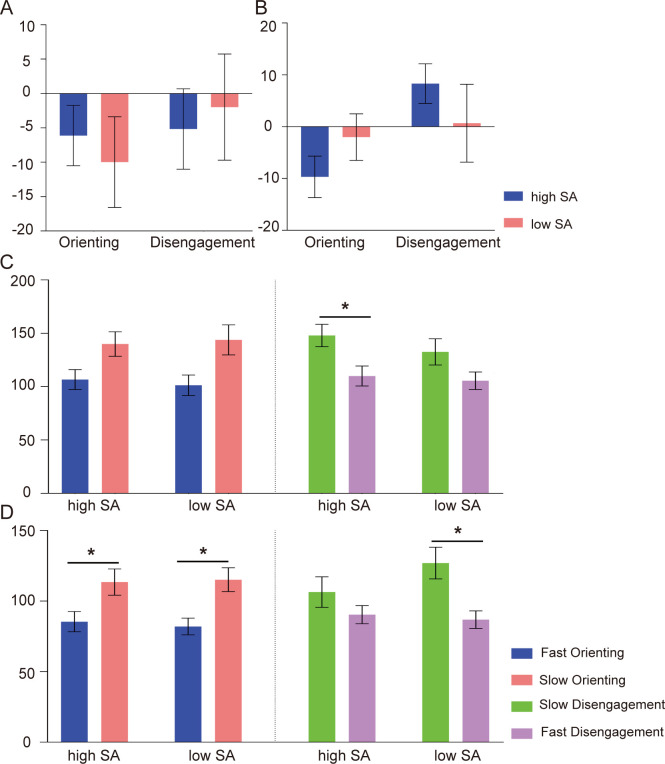
The behavioral results of attentional bias for SN word pairs. The intensities of orienting (N-CON) and disengagement (INCON-N) based on traditional computation in subliminal **(A)** and supraliminal tasks **(B)**. The absolute magnitudes of orienting and disengagement based on response-based computation in subliminal **(C)** and supraliminal tasks **(D)**. high SA: high social anxiety group; low SA: low social anxiety group; *p < 0.05. Error bars indicate standard errors.

For probe RTs, ANOVAs did not reveal any effects of orienting, disengagement, or their interactions with word type and group in either subliminal or supraliminal tasks (*ps* > 0.05). Planned t-tests did not show any significant effects after Bonferroni correction (*ps* > 0.05). The average intensities of orienting (e.g., N-CON) and disengagement (e.g., INCON-N) for SN word pairs in subliminal and supraliminal tasks are shown in **Figures 2A, B**, respectively.

#### 3.1.2 Response-based computation

For the orienting, the ANOVAs revealed larger magnitudes of slow than fast orienting in both subliminal task [F (1,45) = 10.03, *p* = 0.003, $\eta_{p}^{2}$ = 0.18] and supraliminal tasks [F (1,47) = 14.00, *p* < 0.001, $\eta_{p}^{2}$ = 0.22], indicating a greater magnitude of avoidance than vigilance toward negative words. No significant effects were found for other factors or their interactions (*ps* > 0.05). Planned t-tests showed that in the supraliminal task, significant greater avoidance than vigilance was found for SN words in the high SA group [t (24) = 2.71, *p* = 0.048]. This effect was also observed in the low SA group for both NN words [t (23) = 2.85, *p* = 0.036] and SN words [t (23) = 2.94, *p* = 0.028]. In the subliminal task, these effects did not reach significance (*ps* > 0.05). For the disengagement, the ANOVAs indicated larger magnitudes of slow than fast disengagement in subliminal [F (1,44) = 14.1, *p* < 0.001, $\eta_{p}^{2}$ = 0.24] and supraliminal tasks [F (1,48) = 5.94, *p* = 0.02, $\eta_{p}^{2}$ = 0.11], indicating a greater magnitude of disengagement difficulty than fast shifting attention away from negative words. No other main effects or interactions were found (*ps* > 0.05). Following planned t-tests in the subliminal task indicated greater disengagement difficulty to SN words in the high SA group [t (23) = 3.22, *p* = 0.016]. In the supraliminal task, this effect was observed in the low SA group [t (23) = 3.21, *p* = 0.016]. The average absolute magnitudes of orienting and disengagement for SN word pairs in subliminal and supraliminal tasks are displayed in **Figures 2C, D**, respectively.

ANOVAs on the frequencies (trial numbers) did not yield any effects for attentional orienting or disengagement direction in the subliminal task (*ps* > 0.05). In the supraliminal task, no effect of orienting direction was found (*p* > 0.05). Results for disengagement direction indicated that fast disengagement occurred more frequently than slow disengagement [F (1,48) = 4.21, *p* = 0.048, $\eta_{p}^{2}$ = 0.08]. Further planned t-tests indicated that in the low SA group, fast disengagement tended to be more frequent than slow disengagement for NN word pairs [t (23) = 1.87, *p* = 0.07] and SN word pairs [t (23) = 1.91, *p* = 0.07]. In the high SA group, this effect was not observed (*ps* > 0.05).

### 3.2 ERP results

#### 3.2.1 Word pair-elicited N2pc

**Figure 3:**
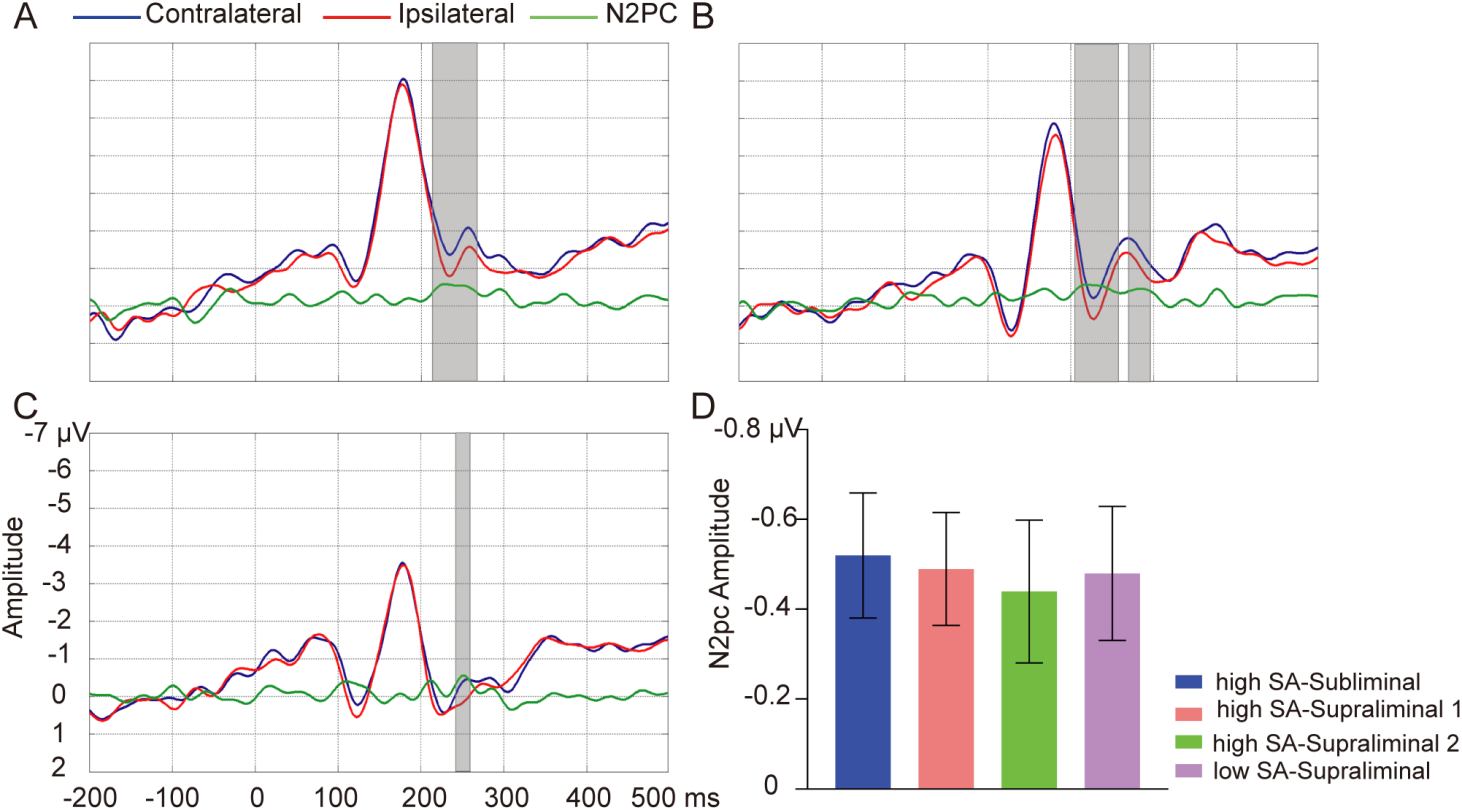
Word pair-elicited N2pc. Grand-average waveforms of SN word pairs on contralateral (blue lines) and ipsilateral sites (red lines) and their difference wave N2pc (green lines) in subliminal task for high SA group **(A)**, in supraliminal task for high SA group **(B)**, and in supraliminal task for low SA group **(C)**. Gray boxes indicate the temporal clusters of significant N2pc effect (contralateral vs. ipsilateral). **(D)** The average amplitudes of N2pc for the significant temporal clusters in subliminal task in high SA group (blue), in supraliminal task in high SA group (light coral for cluster 1, green for cluster2) and in supraliminal task in low SA group (purple). Error bars indicate standard errors.

In the high SA group, the Monte-Carlo permutation tests revealed a significant N2pc in the subliminal task, SN words elicited a larger amplitude on the contralateral site compared to on the ipsilateral site, within the time window of 216-266 ms [T_sum_ = 75.02, *p* = 0.001] (see **Figure 3A**). In the supraliminal task, this N2pc was observed in two clusters in the high SA group: 204-258 ms [T_sum_ = 82.34, *p* = 0.001] and 270-296 ms [T_sum_ = 33.89, *p* = 0.02] (see **Figure 3B**). In the low SA group, the N2pc cluster was found around 242-258 ms in the supraliminal task [T_sum_ = 27.27, *p* = 0.03] (see **Figure 3C**), but not in the subliminal task (*p* > 0.05). The average amplitude of the N2pc for the significant clusters are depicted in **Figure 3D**. No effect was observed for NN words in either group or task (*ps* > 0.05). No group differences in N2pc magnitude were found for any word type (*ps* > 0.05).

#### 3.2.2 Word pair-elicited SPCN

The permutation tests didn’t reveal any significant SPCN for each of SN, NN word type in subliminal or supraliminal task in either group (*ps* > 0.05).

#### 3.2.3 Probe-elicited P3

**Figure 4:**
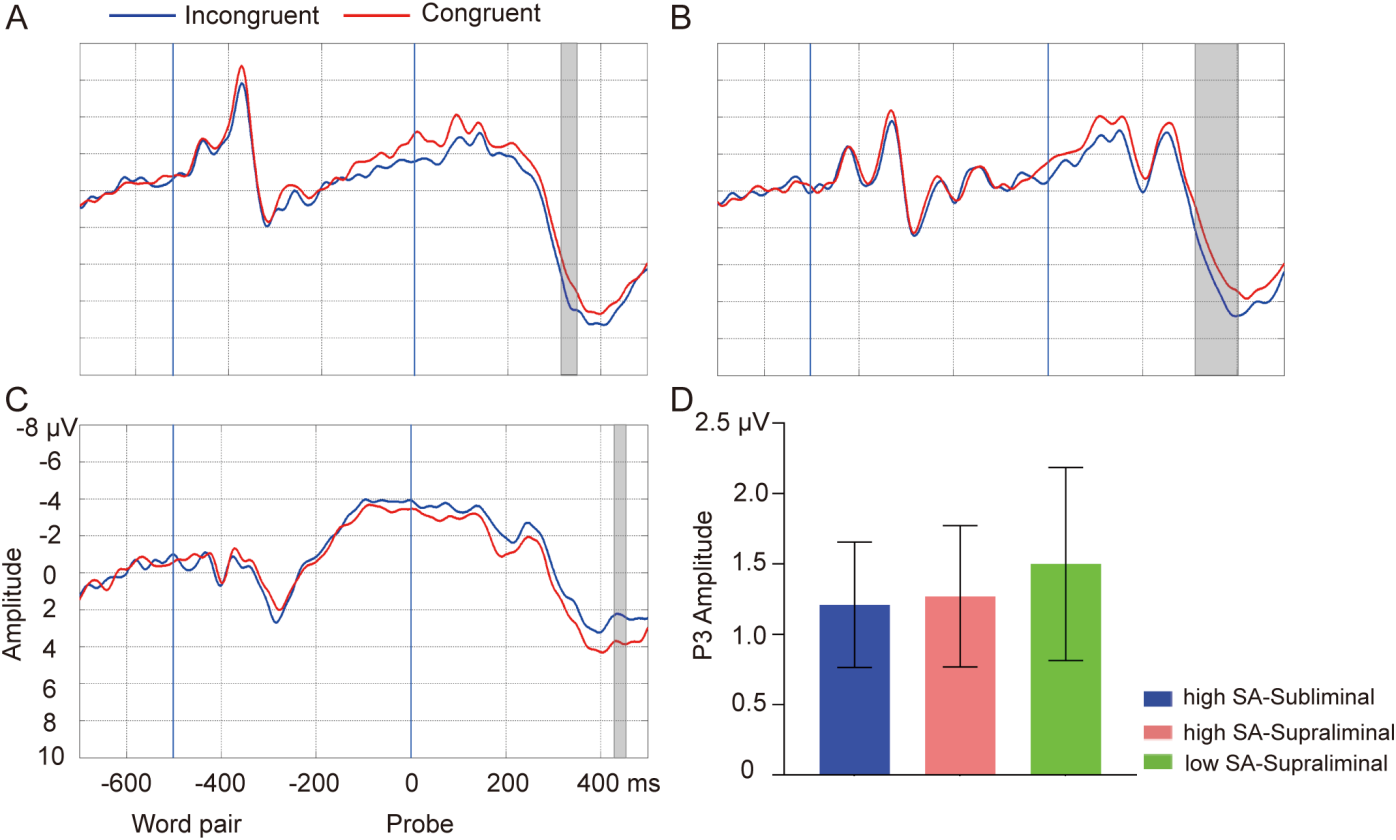
Probe-elicited P3. Grand-average waveforms of incongruent (blue lines) and congruent probes (red lines) in subliminal task for high SA group **(A)**, in supraliminal task for high SA group **(B)**, and in supraliminal task for low SA group **(C)**. Gray boxes indicate the temporal clusters of significant probe effect on P3 (incongruent vs. congruent). The two blue vertical lines indicate the onsets of word pair and probe, respectively. **(D)** The average amplitudes for the significant temporal clusters of probes elicited-P3 in subliminal task for high SA group (blue), in the supraliminal task for high SA group (light coral) and in the supraliminal task for low SA group (green). Error bars indicate standard errors.

In the high SA group, incongruent probes following SN word pairs elicited a larger P3 amplitude compared to congruent probes in the cluster of 316-348 ms on Pz in the subliminal task [T_sum_ = 44.22, *p* = 0.03] (see **Figure 4A**). This effect was also observed around 312-402 ms on Pz in the supraliminal task [T_sum_ = 103.89, *p* = 0.01] (see **Figure 4B**). In the low SA group, an opposite direction of the probe effect was observed, with congruent probes tending to elicit a larger amplitude than incongruent probes following SN words in the cluster of 434-454 ms on Cz in the supraliminal task [T_sum_ = 23.67, *p* = 0.05] (see **Figure 4C**), whereas no effect was found in the subliminal task (*ps* > 0.05). The average amplitudes of P3 effect (INCON-CON for high SA, CON-INCON for low SA) are depicted in **Figure 4D**. No effect was observed for NN words in either group or task (*ps* > 0.05), and no group differences in P3 effect were found (*ps* > 0.05).

#### 3.2.4 Correlation results

**Figure 5:**
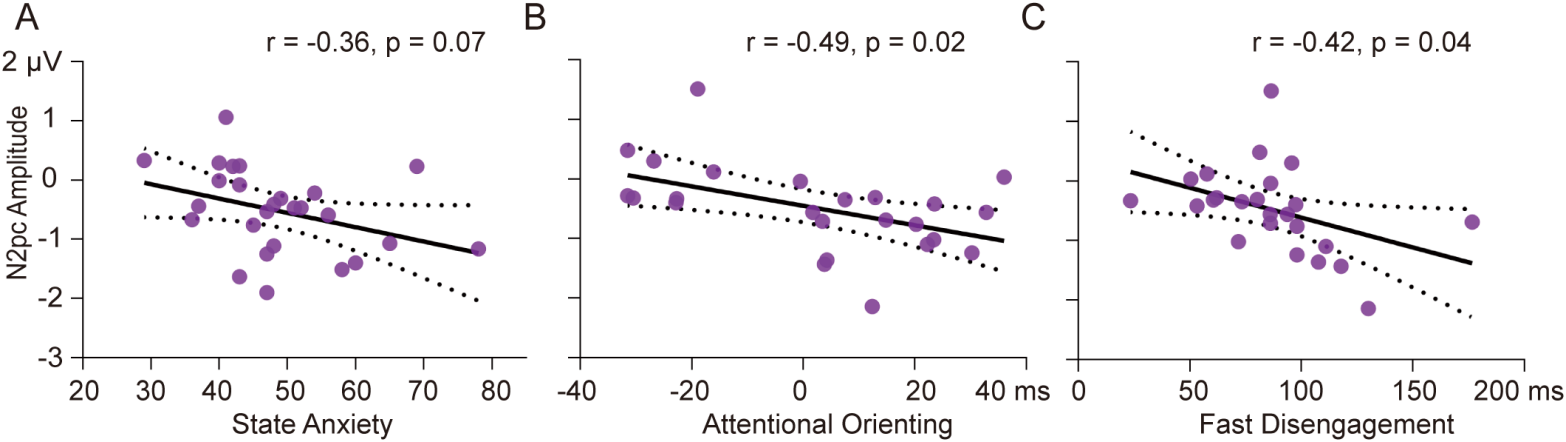
Correlation results. Scatter plots of N2pc amplitude and State Anxiety scores in subliminal task for high SA group **(A)**, N2pc amplitude and behavioral indicator of attentional orienting based on traditional computation in supraliminal task for low SA group **(B)**, N2pc amplitude and behavioral indicator of fast disengagement in supraliminal task for low SA group **(C)**. The black lines indicate the regression lines, and the dashed lines indicate confidence intervals.

Correlation analysis revealed a marginally significant relationship between N2pc amplitude and state anxiety in the high SA group in the subliminal task (r = -0.36, *p* = 0.07) (see **Figure 5A**). Participants with higher levels of state anxiety tended to show greater N2pc amplitudes for subliminal SN words. In the low SA group, N2pc amplitude was related to behavioral indicators of attentional bias mainly in the supraliminal task. Specifically, larger N2pc amplitudes were associated with greater attentional orienting to supraliminal SN words based on traditional computation (r = -0.49, *p* = 0.02) (see **Figure 5B**) and greater magnitude of fast disengagement from supraliminal SN words based on response-based computation (r = -0.42, *p* = 0.04) (see **Figure 5C**).

No significant correlations were found between the intensity of the probe-elicited P3 effect and psychometric measures or behavioral indicators of attentional bias (*ps* > 0.05).

## 4 Discussion

This study used ERP measurements and investigated attentional bias toward subliminal and supraliminal socially negative words at different processing stages in socially anxious individuals. The results showed that, compared to low SA group, high SA group exhibited a significant N2pc effect for subliminal socially negative words, and its amplitude tended to correlate with the severity of anxiety. Additionally, high SA group showed greater parietal P3 amplitude for incongruent probes than for congruent ones following both subliminal and supraliminal socially negative words. These findings suggest that the abnormal attentional bias in social anxiety include preconscious attentional orienting toward social threats at an early stage, as indicated by N2pc, and difficulty disengaging from conscious and unconscious threats at a later stage, as indicated by parietal P3.

For the ERP time-locked to word pairs, the low SA group showed a significant N2pc effect only for supraliminal socially negative words, indicating attentional selectivity and engagement to social threats in healthy individuals (34). Correlation results further supported this by showing that low socially anxious participants with larger N2pc amplitudes had greater behavioral attentional orienting. In addition, we found that N2pc amplitude was associated with fast disengagement, which might suggest that low anxious participants with stronger attentional orienting to negative words tended to shift attention to neutral words more quickly to reduce anxiety. In the high SA group, the N2pc effect was observed not only in supraliminal but also specifically in subliminal presentations, and the amplitude to subliminal socially negative words tended to be related to higher levels of state anxiety. Some studies have proposed that the occurrence of N2pc depends on awareness and task relevancy (63), and it is not elicited when stimuli are presented subliminally (39, 64). But these results were found in healthy controls. Our results indicate that in special samples exposed to their sensitive stimuli or stimuli that trigger symptoms, N2pc can be elicited out of awareness. Several studies have supported this by presenting participants with masked self-faces (38) and presenting smokers with unaware smoking-related images (65). A main symptom of social anxiety is hypervigilance to socially negative feedback; even if patients do not consciously perceive it, attentional bias occurs to motivate behavioral responses such as fleeing from social situations (11, 12). The N2pc to subliminal socially negative words indicates this preconscious attentional engagement and hypervigilance to threats.

In anxiety disorders, unconscious processing of fear-related stimuli is considered a crucial psychopathological factor (4). It is thought to activate fear-related physiological responses before cues are consciously perceived, leading to feelings of uncontrollability and heightened fear (4). The neural basis of unconscious processing operates in a “quick and dirty” fashion. Specifically, information is transmitted along the colliculo-pulvinar-amygdala route. Then the processing in the amygdala influences responses in the fusiform gyrus and extrastriate cortex, facilitating visual perception regardless of awareness (66, 67). N2pc is generated from this extrastriate visual cortex including the parietal lobe, which is responsible for initiating the spatial attention shift to the location of target, and the occipito-temporal areas, which selects relevant information after attention shift (68). The prominent N2pc effect to socially negative words specifically observed in high socially anxious participants and its relationship with anxious symptoms might indicate the pathological processing along this subcortical pathway. The “quick and dirty” processing bypasses the cortex to rapidly initiate an emotional response, so information is not fully analyzed (69, 70). This contributes to hypersensitivity to ambiguous or moderate phobic stimuli (71, 72) and may lead to cognitive symptoms of anxiety, biased or incorrect perceptions of the external world. In comparison, to the supraliminal socially negative words, the processing employed a cortical pathway in both high and low anxious participants (67). The thalamo-cortical pathway relays information from thalamus to the cortex in which the information was analyzed fully and consciously, then transmits information to amygdala, influencing responses in extrastriate visual cortex, where N2pc is generated. Our results suggested that compared to the N2pc at the conscious level, N2pc at the preconscious level was an effective and specific biomarker to indicate the abnormal attentional bias in social anxiety.

For the ERP time-locked to probes, we observed a P3 effect for which amplitude differed between congruent and incongruent probes. The low SA group showed a prominent P3 effect only in supraliminal presentation, with congruent probes tending to elicit larger amplitudes than incongruent ones. It has been found that healthy and low-anxious people tend to avoid negative stimuli (22, 73). The larger P3 amplitude to congruent probes might indicate a compensatory mechanism of top-down executive attention to previously avoided stimuli or location. In subliminal presentation, the P3 effect was not found. This might be because low socially anxious participants were not sensitive to subliminal social threats (13, 74), which was also supported by our behavioral results showing no attentional orienting or disengagement effects for subliminal socially negative words in low SA group. In the high SA group, we found an opposite pattern of probe-elicited P3, that is, incongruent probes elicited larger parietal P3 amplitudes than congruent probes in both subliminal and supraliminal presentations. Previous studies have reported this pattern specifically for probes following angry cues and suggested a disengagement difficulty (75–77). For the high socially anxious participants, their attention was attracted by socially negative words in the early stage. When the subsequent probe appeared in the congruent location, the processing to the direction of the probe was facilitated. But when the probes appeared in the incongruent location, excessive resources and efforts were needed to disengage from the previously attended location of negative word to the task relevant neutral one before processing the probe. Meanwhile, due to the impaired ability of attentional control in socially anxious individuals, they might experience greater difficulty and needed more efforts to achieve attentional disengagement compared to controls (14–16). Thus, a greater P3 amplitude in incongruent trials than congruent ones was observed. This P3 effect reflecting disengagement difficulty in high socially anxious participants might be related with the abnormal activity in attention neural network. According to previous studies, the visual parietal P3 is generated by a distributed network, including the frontal, parietal, limbic, cingulate, and temporo-occipital regions, which are involved in top-down attentional modulation and control according to task goals (78). Compared to healthy controls, anxious individuals exhibit increased activity in these regions (e.g., cingulate) (79), suggesting that they may need to exert greater effort in target-related attentional modulation.

In addition, our study was the first to reveal that compared with low socially anxious individuals, high socially anxious individuals not only had difficulty disengaging from aware social threats, but also from unaware ones, as indexed by the P3 effect to subliminal socially negative words. Previous research manipulating the exposure duration of cues suggested that disengagement difficulty in social anxiety was not observed in brief exposure (e.g., 100 ms), and it depended on awareness of the cues (15, 80). Especially for semantic stimuli, impaired disengagement relied on semantic processing, which required a long duration (81). However, Zajonc proposed that emotions are precognitive (82). Preconscious emotional processing has been found to influence later cognitive activities and emotional states. For instance, subliminally presented emotional stimuli can modulate later character preferences (83) and affect subjects’ emotional states (84). Accordingly, abnormal preconscious processing also produces a cascade of maladaptive effects. In our study, the high SA group unconsciously processed socially negative words, and their attention was automatically captured, which led to a difficulty in disengaging from the locations of these words at a later stage (85).

Our results of the aberrant attentional bias indicated by word pair-elicited N2pc and probe-elicited P3 might be associated with the dysfunction in two attentional systems in social anxiety, as proposed by the Attentional Control Theory (ACT) (16). A bottom-up, stimulus-driven attentional system automatically directs attention to salient or threatening stimuli. This system is housed in the ventral cortical network, including the temporo-parietal junction cortex and the ventral frontal cortex, and modulates visual cortex processing (86). A top-down, goal-directed attentional system voluntarily shifts attention away from task-irrelevant distractors toward targets, relying on the activity of the fronto-parietal network, cingulate opercula, and dorsal prefrontal cortex (79). These two systems frequently interact to direct attention to stimuli in an adaptive way. However, anxiety disrupts this balance, increasing the influence of the stimulus-driven system while reducing the influence of the goal-directed system. This imbalance leads to excessive attention toward threatening stimuli (16, 87) and the impairment in inhibition and shifting of attentional control (88, 89). For socially anxious individuals, this impairment in the balance between the two systems may not only contribute to early-stage orienting and sustained attention to social threats but also lead to difficulties in attentional shifting during later stages. This results in a compensatory strategy, requiring increased effort to reallocate attentional resources according to task goals (16).

The behavioral results suggested that response-based computation was more sensitive in measuring attentional biases in social anxiety compared to traditional computation (62). Traditional computation based on RTs did not reveal any attentional bias, whereas response-based computation uncovered social anxiety related patterns in both attentional orienting and disengagement. The subliminal presentation revealed the disengagement effect specifically in the high SA group, greater magnitudes of slow disengagement compared to fast disengagement. It suggested a dominant pattern of disengagement difficulty from unaware social threats in social anxiety. The supraliminal presentation revealed a tendency of greater avoidance than vigilance in both the high and low SA groups. This is consistent with the temporal properties of attentional bias, that when threats presented for 500 ms, individuals switch to avoidance, after initial automatic vigilance (90). The disengagement effect was also found in supraliminal presentation, greater disengagement difficulty compared to fast disengagement, was found in the low SA group; however, since they also tended to have higher frequencies of fast disengagement, the magnitude results were counteracted. Overall, the behavioral measurement showed consistent results with ERP. The attentional bias to the socially negative words distinguished participants with high and low social anxiety mainly at the unconscious level, rather than at the conscious level.

Our finding might have clinical implications. Firstly, it supported that the cue-elicited N2pc and probe-elicited P3 in a dot probe task to unconscious social threats could potentially serve as auxiliary indicators to predict the susceptibility to social anxiety in the general population or assessing the symptom severity in individuals with social anxiety disorder. In addition, attention bias modification (ABM) training is widely employed to treat social anxiety disorder. In ABM, threats and neutral stimuli are concurrently presented consciously (i.e., 500 ms), the following targets are designed to appear more frequently to the location of neutral stimuli, to help patients learn biasing attention away from threats (91). Our results suggested that the unconscious bias to threats is an important pathological factor of social anxiety, and it might support a new variant of ABM. That is, presenting threat-neutral stimuli pairs unconsciously (i.e., 20 ms or less), and guiding patients’ unconscious bias away from threats might also help alleviate symptoms.

There are several limitations in our study. First is the sample size of 50 for the two groups was relatively small. Increasing the sample size in future research would enhance the statistical power and generalizability of our findings. Second, the valences and arousals between socially and non-socially negative words were not strictly matched. Attentional bias-relevant N2PC and P3 effects were found only in socially negative words but not in non-socially negative words, it might due to their differences in emotional dimension rather than in social dimension. Third, we cannot ensure the unawareness of subliminally presented word pairs across all the participants who completed the EEG experiment. Although we verified the unawareness of subliminal exposure in a separate group, the individual variability in sensory threshold exists. Participants with lower threshold may have been aware of the subliminally presented words. Finally, our findings were obtained in Chinese participants. Previous studies have shown culture differences between east Asians and Westerners. East Asians tend to view themselves in relation to others and exhibit heightened sensitivity to others’ feelings and evaluations, while Westerners tend to view themselves independent from other (92). This difference might contribute to different cognitive processing bias (93, 94) and different prevalence of social anxiety (95) between these cultures. Therefore, whether our findings can be generalized to Western participants requires further examination.

In summary, our results indicate that compared to low socially anxious individuals who exhibited attentional bias only at the conscious level, high socially anxious individuals also demonstrated attentional bias at the preconscious level. They exhibited preconscious attentional orienting to social threats, as indexed by N2pc, and difficulty in disengaging from conscious and unconscious social threats, as indexed by parietal P3. These ERP indicators reveal the temporal properties of attentional bias in social anxiety, and emphasize the importance of early automatic attentional orienting and later disengagement difficulty as two key factors in understanding the psychopathology of social anxiety (17).

## Data Availability

The raw data supporting the conclusions of this article will be made available by the authors, without undue reservation.
