# Supplementary material for: Electrocortical signatures of attentional bias toward subliminal and supraliminal socially negative words in social anxiety
Source: Front Psychiatry. 2025 Feb 13;16:1506516. doi: 10.3389/fpsyt.2025.1506516 (PMC11865052; doi:10.3389/fpsyt.2025.1506516)
Supplement: Supplementary file 1 [file Table1.docx]

Supplementary Material

# Table S1.

The Chinese two-character words used in the dot-probe tasks and their English translations.

| Socially Negative Words | | Non-socially Negative Words | | |  |
| --- | --- | --- | --- | --- | --- |
| 笨蛋 | Idiot | 变质 | Go bad |  |  |
| 懦夫 | Coward | 车祸 | Car accident |  |  |
| 忽视 | Ignore | 摧毁 | Destroy |  |  |
| 诅咒 | Curse | 倒闭 | Close down |  |  |
| 鄙视 | Despise | 盗窃 | Steal |  |  |
| 傻子 | Fool | 地狱 | Hell |  |  |
| 失败 | Failure | 妨碍 | Hamper |  |  |
| 处罚 | Punish | 废弃 | Obsolete |  |  |
| 开除 | Expel | 锋利 | Sharp |  |  |
| 奚落 | Taunt | 腐烂 | Rot |  |  |
| 窝囊 | Wimp | 干枯 | Dried-up |  |  |
| 丢人 | Shameful | 棺材 | Coffin |  |  |
| 欺负 | Bully | 寒冷 | Freezing |  |  |
| 孤立 | Isolate | 毁灭 | Ruin |  |  |
| 厌恶 | Detested | 混乱 | Chaos |  |  |
| 责怪 | Blame | 疾病 | Illness |  |  |
| 排斥 | Ostracize | 简陋 | Tatty |  |  |
| 嘲笑 | Jeer | 枯萎 | Wither |  |  |
| 质问 | Oppugn | 枯燥 | Boring |  |  |
| 批评 | Criticize | 落后 | Backward |  |  |
| 蔑视 | Disdain | 泥泞 | Miry |  |  |
| 可笑 | Ridiculous | 溺亡 | Drowned |  |  |
| 辱骂 | Abuse | 破败 | Dilapidated |  |  |
| 过错 | Fault | 破产 | Bankrupt |  |  |
| 尴尬 | Awkward | 抢劫 | Rob |  |  |
| 难堪 | Embarrassed | 丧失 | Lose |  |  |
| 愚蠢 | Stupid | 沙哑 | Hoarse |  |  |
| 混蛋 | Bastard | 衰退 | Recession |  |  |
| 拒绝 | Refuse | 死刑 | Death penalty |  |  |
| 笨拙 | Clumsy | 损毁 | Damage |  |  |
| 仇恨 | Hatred | 吞噬 | Engulf |  |  |
| 偏见 | Prejudice | 危急 | Desperate |  |  |
| 畏缩 | Cower | 污浊 | Dirty |  |  |
| 羞愧 | Ashamed | 腥臭 | Stinking |  |  |
| 耻笑 | Sneer | 血腥 | Bloody |  |  |
| 敌视 | Hostile | 有毒 | Toxic |  |  |
| 侮辱 | Insult | 杂乱 | Mess |  |  |
| 不满 | Dissatisfied | 灾难 | Disaster |  |  |
| 捉弄 | Tease | 造假 | Counterfeit |  |  |
| 无能 | Incompetence | 窒息 | Suffocate |  |  |
| Neutral Words | | | | | |
| 银行 | Bank | 平均 | Average | 工业 | Industry |
| 机构 | Institution | 初步 | Preliminary | 秩序 | Order |
| 主题 | Theme | 深沉 | Profound | 日常 | Daily |
| 答案 | Answer | 文学 | Literature | 技术 | Technology |
| 对策 | Strategy | 机场 | Airport | 主力 | Main force |
| 微妙 | Subtle | 依旧 | Still | 任务 | Task |
| 确认 | Confirm | 测量 | Measurement | 人类 | Human |
| 强调 | Emphasize | 不同 | Different | 适度 | Moderate |
| 遵守 | Comply | 简单 | Simple | 提醒 | Remind |
| 说明 | Illustrate | 书籍 | Book | 对称 | Symmetry |
| 大概 | Approximate | 观念 | Concept | 储存 | Storage |
| 考察 | Inspect | 真相 | Truth | 景色 | Scenery |
| 世界 | World | 主要 | Main | 销售 | Sale |
| 标志 | Sign | 俨然 | Seemingly | 视野 | Vision |
| 现实 | Reality | 申请 | Application | 听力 | Hearing |
| 采纳 | Adopt | 挖掘 | Excavate | 职业 | Profession |
| 编辑 | Edit | 调整 | Adjust | 散发 | Emit |
| 平缓 | Smooth | 关键 | Key | 国家 | Country |
| 筹划 | Plan | 治理 | Govern | 中立 | Neutral |
| 语言 | Language | 调节 | Regulate | 正规 | Formal |
| 方法 | Method | 信息 | Information | 建造 | Build |
| 铁路 | Railway | 工人 | Worker | 接收 | Receive |
| 新闻 | News | 配合 | Cooperate | 阐述 | Expound |
| 解释 | Explain | 维持 | Maintain | 几率 | Probability |
| 细节 | Detail | 常规 | Routine | 法规 | Statute |
| 零星 | Scattered | 普通 | Common | 贸易 | Trade |
| 房屋 | House | 凑巧 | Coincidence | 义务 | Obligation |
| 传统 | Tradition | 货币 | Currency | 全球 | Global |
| 举办 | Hold | 事务 | Affair | 光线 | Light |
| 尺寸 | Size | 整理 | Tidy | 城市 | City |
| 公寓 | Apartment | 彻底 | Thorough | 科研 | Research |
| 开支 | Expenditure | 事物 | Thing | 职责 | Duty |
| 深入 | Deep | 填补 | Fill | 含蓄 | Implicit |
| 图表 | Chart | 证实 | Verify | 风格 | Style |
| 开展 | Carry Out | 默默 | Silently | 寻觅 | Seek |
| 严密 | Rigorous | 叮嘱 | Enjoin | 悬挂 | Hang |
| 工作 | Job | 差异 | Discrepancy | 参观 | Tour |
| 列车 | Train | 状态 | State | 电脑 | Computer |
| 平凡 | Ordinary | 覆盖 | Cover | 分配 | Allocate |
| 思想 | Thought | 观望 | Wait and see | 播放 | Broadcast |
